# Controversy and Growth Points in the Activity Theory in Psychology

**DOI:** 10.11621/pir.2021.0401

**Published:** 2021-08-15

**Authors:** Andrey D. Maidansky

**Affiliations:** a Belgorod National Research University, Belgorod, Russia; b Institute of Philosophy, Russian Academy of Sciences, Russia

**Keywords:** Activity, action, affect, *perezhivanie*, freedom

## Abstract

**Background:**

Activity theory is the most powerful and influential current of Russian psychology in the world today. It considers the psyche to be a special form or function of object-oriented activity. The level of psychical development of a living being is directly proportional to the variety and freedom of its activities.

**Objective:**

The aim of this article is to explore the key growth points in activity psychology through the analysis of arguments among its creators — S.L.Rubinstein, L.S.Vygotsky, A.N. Leontiev, and P.Ja. Galperin. Vygotsky dreamed of building a scientific psychology on the model of Marx’s *Das Kapital*; his project is resumed in this article.

**Results:**

The author traces how, due to Walter Cannon’s experimental research, Vygotsky came to the activity concept of affect, in which he finds the primary “cell” of the psyche. The problem of the relationship between concept and affect became the central problem of his “acmeistic psychology.” While Vygotsky focused on the affective reflection of activity in the subject, Leontiev focused on its cognitive side, directed toward the object. In the objective world, the psyche serves a person’s material life-activity, performing a search-and-orientation function. Leontiev considered consciousness a structural projection of that activity, but Galperin argued that Leontiev never managed to overcome the dualism of consciousness and activity.

**Conclusion:**

A new path to the realization of Vygotsky’s dream is outlined. The proposed solution is based on Spinoza’s concept of affect and the idea of freedom, interpreted as “the affect in the concept.” (Vygotsky)

## Introduction

Marxism has always claimed to be a theory of action. Its cornerstone is the concept of labor, practice, or activity, understood as the process of transforming both the external world and human beings themselves. Naturally, Marxist psychology, since its birth, has also declared that the psyche^1^ is derived from activity, *i.e*., that it is a special form or function of object-oriented activity.

Already at the beginning of 1926, L.S. Vygotsky formulated the key postulate of the psychological theory of activity: “Mind (*psikhika*) is the formation of something stable amidst the streaming. It is a selection organ, a sieve, which changes the world so that we can act.” (2018, p. 92)

Vygotsky then criticized the widespread materialistic notion of psyche as a mere reflection of objective reality. A thermometer also reflects something real; it is important to understand *what and how* the psyche reflects. It is a selective reflection: only what is *valuable to the activity* is fixed as a psychical phenomenon. If everything were reflected in the psyche indiscriminately, as in a mirror, then it would be impossible to orient oneself in the limitless and chaotic sensory stream, and to find what is required for life. Activity would be blind. “If we would see (be conscious of) everything, we would see nothing.” (Stern) The psyche discerns and singles out the stable, the self-identical, “distorting reality to the advantage of the organism,” and each sense organ reflects the world with its “coefficient of specification.” (Vygotsky, 2018, p. 92)

At first, Soviet psychologists, feeling sympathy for American behaviorism, tended to blend the concepts of activity and behavior. Thus, the first edition of the *Great Soviet Encyclopedia* had no entry for “Activity,” but a huge article on “Behaviorism” was commissioned from John B. Watson “in view of the novelty of the topic and the great interest he aroused among modern scientists, including Marxists.” (Watson, 1927, p. 434)

There was also no entry for “Activity” in B.E. Varshava and L.S. Vygotsky’s *Psychological Dictionary* (1931). At the same time, however, Vygotsky and other Soviet psychologists were beginning to develop their variants of activity theory. Vygotsky dreamed of “creating our *own Das Kapital*” for psychology (1997b, p. 330; 2018, p.87). No more, no less. Let us see how far he and his school succeeded in this task.

The aim of this article, though, is not so much to tell the story of the past as to define the weak spots inside activity theory; they are, at the same time, its *growth points*. These promising flaws are best seen as forks in the road of thought, occurring in the course of polemics and mutually critical attacks undertaken by the creators of activity psychology. We should listen to their dialogue and try to participate in it. The archives that have been opened in recent years can help us here. In them, we see that the disputes were conducted frankly and without undue politesse. Many discrepancies and arguments between the parties have not previously appeared in print. For example, the recently published volume of Vygotsky’s notebooks contains previously unknown materials about his polemics with Aleksei Leontiev. Formerly, we only knew about that “schism,” which decided the future of activity psychology, from Leontiev himself.

### S.L. Rubinstein versus A.N. Leontiev: the Internal and the External in Activity

Sergei Rubinstein started to develop the concept of activity back in 1922, asserting that activity creates its own subject. He proposed to place this principle of “creative self-activity” at the foundation of pedagogical practice (Rubinstein, 1989).

In his *Fundamentals of General Psychology* (1940), Rubinstein described a scheme of activity in which a certain “internal change” is located between stimulus and response. This “internal change” includes all the processes of life-activity and bodily states that affect particular actions. It is just this factor that makes the active reaction of a living being different from mechanical reactions in inanimate nature. Even the simplest organism can react differently to the same external stimuli, depending on its internal state at the time. “The higher the level of development, the greater the role played by internal conditions.” (Rubinstein, 2003, p.126)

Spinoza called the internal state of a living body which influences its ability to act, “affect.” But Spinoza, unlike Rubinstein, understood that any internal change is an *effect, a reflection of the external activity* of this body. In the absence of external activities, there are no internal changes. That is why God-substance, acting only on itself, does not change and has no affect.

In 1959, a year before his death, Rubinstein criticized the cultural-historical theory of interiorization developed by Leontiev and Galperin for not taking into account how cultural schemes of activity are refracted through the prism of “initial internal preconditions in the individual.” These are “organic, natural, in particular physiological, conditions,” *i.e.,* genetically inherited bodily structures and innate automatic reactions. In the historical development of mankind, they “play an invariable, that is *constant*, role.” (Rubinstein, 1973, p.223)

Since man stands at the top of the evolutionary ladder, his activity should depend on these natural factors as much as possible, given that “the higher the level of development, the greater the role played by internal conditions.” At the same time, Rubinstein declared *social, cultural* schemes and norms of activity to be *external* conditions of human development. The process of their interiorization was interpreted by Rubinstein as the “determination of abilities from the outside.”

A.N. Leontiev, for his part, argued that the subject’s body itself is formed by object-oriented activity, both its morphology and brain structure, and how they react to external stimuli. The empirically observed dependence of activity on the structure of the body is, in fact, the dependence of the *current* act of activity on its own *previous* acts which formed this or that bodily structure. The “internal” and the “external,” subject and object, are not preconditions of activity, but *its extreme poles*. In the process of activity, the “external” and “objective” are transformed into the subjective, and vice versa. The very person, the “particular subject,” is presented here as “the *inner moment of activity*. The category of activity now comes to light in its actual fullness, as embracing both poles— both the pole of object and the pole of subject.” (Leontiev, 2004, p.122)

Leontiev embraced the activity principle far more profoundly and more consistently than Rubinstein. It is wrong to consider interiorization of cultural forms of activity as a determination of the psyche from *outside*, he said. After all, culture is created by activity and constitutes the objectively tangible form of its own being. The process of interiorization simply means a *change of activity form,* the re-appropriation and de-objectification of what was previously “posited” by the activity itself as an artifact of culture.

Higher, specifically human activity is joint, collective activity. It always occurs in society as an internal, immanent condition of human life. Among the external, “prehistoric” conditions of *human* activity and mind are body morphology, innate reactions, and everything that was formed in the processes of *animal* activity, all that was not created by human labor.

Rubinstein mistakenly took natural factors for “the internal” and, vice versa, regarded the real internal (cultural, specifically human) as something external to the human mind. Hence his reproach of the “mechanistic nature of this [cultural-historical] interpretation of personality and the development of its abilities, since the very activity of the subject is thought to be determined *only* by the object, *only* from the outside.” (Rubinstein, 1973, p. 227)

Here Rubinstein understands the “subject” as an *individual*, and the “object” as any thing *outside* this individual, regardless of whether it is a natural thing or an artifact. For cultural-historical theory, this difference is extremely significant. The world of artifacts belongs to the subject and forms the “inorganic body” of the human mind. The true subject is not an individual organism with its genes and reflexes, but a human community, *i.e*., a circle of people sharing a common body of culture. Every single “higher psychological function,” in Vygotsky’s terms— *i.e.,* every specifically human mode of mental activity— is of social origin. The individual acquires such functions only by way of cultural communication with other humans.

The alternative to the cultural-historical stance is the individualistic stance: a particular body and psyche vs. the outside world. Marx called the view of the world from the standpoint of abstract individual a “Robinsonade.” In psychology, such a Robinsonade is an illusion as natural as believing in the rotation of the Sun around the Earth. In the course of their polemics, A.N. Leontiev bypassed this foundational feature of Rubinstein’s criticism of the concept of the interiorization of higher psychological functions and corresponding human “abilities.”

Both Rubinstein and Leontiev knew very well that any activity is always *reflected* in its subject in its current state. It remained to realize that the psyche is nothing but this reverse reflection of activity, or its “reflection into itself,” as Hegel would say.

All activities end in a double result: on the one hand, the form of the external object changes, and on the other, there is a change in the state of the acting subject. The change that subjects themselves undergo in the process of object-oriented activity Spinoza called “affect,” with the caveat that the class of affects includes only those changes which influence the individual’s activity potential, or “the affections of the body by which the body’s power of acting (*agendi potentia*) is increased or diminished, aided or restrained.” (*Ethics*, III, def.3)

At this point Vygotsky took the baton from Spinoza. “Affect is the alpha and omega, the first and last link, the prologue and epilogue of all mental development.” (1998, p.227)

From then onward, the psychology of activity forked into two branches. Vygotsky focused on the affective reflection of activity in the subject, while Leontiev focused on the cognitive side, directed towards the object; specifically, he concentrated on the search and orienting function that the psyche fulfills in the objective world.

### L.S. Vygotsky: Affect in the Structure of Activity

Marx wrote that the history of industry was “the exposure to the senses of human *psychology*.” In Vygotsky’s eyes, another open book of human psychology is *art*, especially theater.

In his early manuscript *The Psychology of Art*, the principle of object-oriented activity is not formulated and plays no significant role. The whole study revolves around the concepts of emotion and affect .The activity of imagination is defined as the “discharge of affect,” art as the work of “very special emotional thinking.” And the collision of affects, or “affective contradiction,” forms the “true psychological basis for our aesthetic response.” (Vygotsky, 1987b, pp. 48-49, 138) At the same time, the *activity nature* of affect, and of the psyche in general, remains on the far side of the Moon.

However, even earlier, and already in his first major work *Educational Psychology*, Vygotsky declared that “an emotional reaction is a powerful guide of behavior. It is in an emotional reaction that the activity of our organism manifests itself. ... At every turn, the emotions act as the ruler of behavior.” (Vygotsky, 1997a, p.102) “The transition to a psychical type of behavior undoubtedly occurred on the basis of the emotions,” he adds. The natural cycle of psychical activity begins with the affect of desire and ends with the affects of pleasure and displeasure. These emotional reactions, “which arise earlier than all the other reactions, are the primary forms of a child’s purely mental behavior.” (*Ibid*., p. 103)^2^

In August 1930, the journal *I Want to Know Everything* published a small article entitled “The biological basis of affect,” written by Vygotsky in response to the question from a “group of readers.” The major portion of it was a popular retelling of Walter Cannon’s *Physiology of Emotions*.^3^ Its central thesis ran as follows: in animals, affect serves “basic life instincts,” preparing the organism for activity. The major emotions have an “energizing effect” upon the organism, releasing its inner “reservoir of power.” (*cf., agendi potentia*)

Vygotsky could not help but recognize in this thesis Spinoza’s definition of affect as a state of the body that increases and aids (as in Cannon’s experiments), or diminishes and restrains, (as in Luria’s “combined motor method” experiments or in Freud’s clinical practice with neurotics) the body’s power to act.

But Vygotsky sought to distinguish between animal and human affects. The “progressive development of emotions” consists in replacing innate reactions by *ideas*. “What Cannon demonstrated was that it is not the emotions themselves that die away, but only their instinctive component. The role of the emotions in the human mind is different [than in animals]. They are isolated from the instinctive domain and transferred to an entirely new plane” (Vygotsky, 1987a, p.332). This is the ideal, the cultural plane.

With this, an entirely new problem arises— the issue of the relationship between idea and affect. In the course of trying to solve this problem, Vygotsky years later, in 1932, turned again to the art of theater. The actor is a professional creator of affect, trying to bring the viewer to the point of the “highest emotional shock,” he argued. In doing so, what happens in the actor’s own soul?, Diderot asked. Vygotsky’s answer: it depends entirely on his *culture*, the world of ideas in which the actor’s soul is immersed. Theatrical affects are *ideal*; they don’t reflect and represent organic processes in the actor’s body, but in people’s social life.

“These are idealized passions and movements of the soul; they are not natural, live feelings of one actor or another; they are artificial; they are created by the creative force of man and to that extent must be considered as artificial creations, like a novel, a sonata, or a statue.” (Vygotsky, 1999, p.239)

Vygotsky called the idealized cultural emotion *perezhivanie*, effectively equating these two terms: “The experience (*perezhivaniya*) of the actor, his emotions....” (*Ibid*., p.244) The tower of human consciousness is built of “bricks” of *perezhivaniya*. But, unlike bricks, *perezhivaniya* are fluid and changeable; these “soul movements” are able to change their meaning during life, including under the influence of theatrical acting, or reading a novel or poem.

Emotional *perezhivaniya* get their meanings from *ideas*. The culture of feelings is to *idealize passions*, that is, to subordinate natural affects to the highest goals of social life, and to teach peopleto 1) induce the required affect, and 2) change the “order and connection” of their emotions. Emotion is embedded as a dynamic element into a certain (historical) system of ideas. This is “the path to mastery of emotions, and, consequently, the path of voluntary arousal and artificial creation of new emotions. [...] Only indirectly, creating a complex system of ideas, concepts, and images of which emotion is a part, can we arouse the required feelings.” (Vygotsky, 1999, p.243)

In recent years, the notion of *perezhivanie* has come to the forefront in Vygotsky studies. In his lectures on child psychology, *perezhivanie* was defined as a “dynamic unit of consciousness.” It is the internal relationship of a person to things and events of the external world, which includes attention, thinking, and emotions, and contains “all the basic properties of consciousness.” (Vygotsky, 2001, p. 213.

A.N. Leontiev regarded the turn to the study of consciousness and *perezhivanie* as a departure from activity theory. But in fact, Vygotsky’s notion of *perezhivanie* was a further development of the activity concept of affect. Vygotsky vindicated “the understanding of affect as an integral psychophysiological reaction that *includes in itself experience* [*perezhivanie*] *and behavior* of a certain type and represents a unity of the phenomenal and objective sides.” (Vygotsky, 1999, p. 159; *emphasis added*) Commenting on Gregorio Maranon’s experiments, Vygotsky wrote of an “internal interweaving of experience [*perezhivanie*] and the organic reaction in the composition of affect.” (*Ibid*., p. 93)

Thus, *perezhivanie* is a social affect, observed from its internal, or “phenomenal” side. And human consciousness is a system of *perezhivaniya* as the “idealized passions and movements of the soul.”

### L.S. Vygotsky: the Psychology of Freedom

Already in January 1924, in his paper at the Congress on Psychoneurology, which opened up his pathway into Big Science, Vygotsky posed the problem of “liberation from the most terrible slavery, the slavery to oneself, and from the most bitter dependence, the dependence on one’s own nerves and psyche.”^4^ This is how he translated Spinoza’s statement about freeing a person from slavery to his affects into a language familiar to psychoneurologists.

To the extent that a man subdues and controls his own affects, he becomes master of his behavior and mental life. An infant, like an animal, is a slave to its natural desires. The adoption of cultural norms of behavior, ideas, always involves “moderating and restraining affects” (Spinoza). From the material of natural affects, people create artificial emotions, *perezhivaniya*. Natural affects are moderated and restrained by cultural ones. How this happens is seen clearly already in children’s games. Every rule of a game is an idea. Games give the child the first experience of the self-reliant regulation of his or her affects through ideas.

While natural affects serve the body’s vital activity, cultural affects serve the activities of society. These are the states of the collective “quasi-body” (Spinoza) or “inorganic body” (Marx) of mankind. Being “grown” into the psyche of an individual, they allow him to emotionally experience (*perezhivat’*) things that are useless, if not harmful, in a biological respect, but valuable to society.

Behind every cultural emotion there stands an *idea*— a norm or scheme of social activity. Ideas are assimilated (interiorized) through affects, together and simultaneously with affects. If an idea did not get the slightest emotional response, it simply would not be grasped. The soul would remain deaf to it.

Ideal emotions rebuild the biological system of affects, establishing a cultural order and connection between them. “Like all other mental functions, emotions do not remain in the connection in which they are given initially by virtue of the biological organization of the mind. In the process of social life, feelings develop, and former connections disintegrate; emotions appear in new relations with other elements of mental life, new systems develop, new alloys of mental functions and unities of a higher order appear within which special patterns, interdependencies, special forms of connection and movement are dominant. To study the order and connection of affects is the principal task of scientific psychology.” (Vygotsky, 1999, p.244)

Science should help a person tame the “wild” affects by organizing them intelligently, *i.e.,* in accordance with the order and connection of ideas. This is exactly the same thing that art does, only by other means. Both scientific psychology and art solve the problem of rational management of the stream of *perezhivaniya*; they both aspire to liberate the soul from the natural slavery to affect.

Vygotsky saw the key to the solution in the *concept*. Brought to the light of consciousness, *conceptualized* affect ceases to be a slave, or passive bodily state. “The affect in the concept becomes active” (Vygotsky, 2018, p.410). “To understand the affect is an active condition and is *freedom*. *Freedom*: the affect in the concept.” (Vygotsky, 2018, p.209)

Hence, art is an *exercise in freedom*. Art puts affect at the service of concept. Here, the human mind learns to command passions and to direct its feelings towards higher, ideal goals. This process of the psychological liberation of the personality is the subject matter of practical training for the artist, and the subject of theoretical research for the psychologist.

Art and scientific psychology have the same subject matter and solve the same problem. Vygotsky came close to this idea but did not formulate it directly. In *The Psychology of Art*, art was seen as a kind of affective vaccination, allowing us to develop immunity to the passions of real life and, thus, to acquire psychological “superhealth.”

The “acmeistic psychology,” or “height psychology,”^5^ that Vygotsky intended to create, can be defined as the *psychology of freedom*. This is a theory of forming a “self-active free person” (*samodejatelnaja svobodnaja lichnost*). That is what Spinoza taught us. “He all the time investigates the question as to how the *motion* toward freedom *really* takes place: toward *a life guided by reason*— and this is freedom. His central idea is the power of reason.” (Vygotsky, 2018, p.209)

“Spinoza’s theory *implicite* contains the whole acmeistic psychology, the whole theory of concepts, affects and volition, the semantic and systemic structure of consciousness, which we *explicite* developed. Spinoza has the *idea* of man, which can serve as a model for human nature: This makes his theory of the passions the prolegomena for a psychology of man.” (Vygotsky, 2018, p.375)

Both Spinoza and Vygotsky sought to teach man to think and live freely; both saw the purpose of their science as increasing man’s activity potential and degrees of freedom.

In outlines written during the last years of his life, Vygotsky drew a plan of a three-storied building of human psychology:

1. “the direct movement from life to consciousness”;
2. “the inner reality,” the realm of consciousness, inhabited by *perezhivaniya*, *znacheniya* (meanings), and *smysly* (purposes), where “communication with oneself ” goes on; and
3. “the reverse movement from consciousness to life (consciousness changes life).” (Vygotsky, 2018, pp. 354–355)

The first floor was the fiefdom of instrumental psychology. Here, the “external in-growing (of the *sign*)” takes place; Vygotsky and his team had been investigating this process since the mid-20s.

Next came the “internal ingrowing (of *meaning*)”; this was the topic of his book *Thinking and Speech*.

Construction of the third floor was just begun in *The Teaching about Emotions*. The process of a person’s transition to free, rational conscious life can be called the *exteriorization of consciousness*.

### A.N. Leontiev and P.Ja. Galperin: Looking for the Nature of Psyche

Leontiev departed from Vygotsky’s concept early on, before reaching his “second floor.” Moving into the “inner reality” of consciousness seemed to him a betrayal of the activity approach. He wished to continue studying consciousness as a form of human objective-practical activity. “Seek the consciousness of man here, in the objective world!” (Leontiev, 1994, p.39)

For Vygotsky, that approach defined only the initial stage of research, which had already passed. Yes, *in the beginning* was the Act, but then the Act *became the Word* and gave rise to consciousness. “The meaningful word is a microcosm of human consciousness.” (Vygotsky, 1987c, p.285) Now he starts to analyze of how purposes (*smysly*) are formed in consciousness.

Meanwhile, Leontiev drew cultural psychology back to external activity, in the bosom of which consciousness was born. Vygotsky commented that: “Development is ignored. Everything is moved to the beginning. But then everything [is to be moved] to the conception. The most important thing does not take place in the beginning, but in the end, for the end contains the beginning. The height (*vershinnaja*) viewpoint. [We] should not work near the lower boundaries all the time.” (2018, p.247)

The transition from life practice to consciousness was only the first floor of scienti fic psychology. Research should not remain stuck at this early stage, “near the lower boundaries.” Furthermore, consciousness should be investigated as such, in its inner reality, and then in its outer, practical implementation. The “height/acmeistic” subject matter of psychology is *conscious life*, or what is the same, *human freedom*.

“The direct movement (from life to consciousness) is only important to the extent that it allows us to understand the *reverse movement* from consciousness to life (consciousness changes life), the dependency of life on consciousness.” (Vygotsky, 2018, p.355)

Leontiev could not help but respond to his teacher’s challenge. In the 1930s, he went back to the subject of emotions.^6^ One of the sections of his doctoral thesis was devoted to this topic. The work was conceived as the first volume of his monograph *Development of the Psyche*; it has not been published, but some key statements are known from Piotr Galperin’s letter of October 1940 to Leontiev (Galperin, 1997). Leontiev explained the problem of linking affect and intellect, bequeathed by Vygotsky, through the relationship of activity and action. Here, affect was quite rightly defined as “the internal representation of activity.” Galperin called this definition “deep and important” and approved the concept of psyche as the “internal form of activity” derived from the external activities that take place in the physical world.

However, he continued, “precisely the understanding of psyche as activity remains undeveloped [in Leontiev’s book]. It is more likely postulated and applied in a broad genetic construction than it is revealed and substantiated as such. And this leads to the replacement of psyche as activity by psyche within activity, psyche standing behind activity and remaining a set of phenomena and *perezhivaniya* of consciousness as before.” (Galperin, 1997, p.4)

In other words, Leontiev failed to *derive* the inner world of consciousness from the external, object-oriented activity. But this was the main point of his theoretical program, as opposed to Vygotsky’s program. Leontiev’s closest associate insisted that the problem remained unsolved.

“In fact, in the scheme you sketched, there stands out clearly a parallelism of consciousness and behavior. In behavior— activity, action, operation; in consciousness— affect, purpose, meaning. [...] Consciousness represents and reproduces, in its own language, the plot of actions and things. It is true that activity is mediated by `reflection’ and is one with it. But what kind of activity? External, non-psychological activity! *And when we are asked what **psychical** activity itself is*, it turns out to be affect, purpose, meaning, *perezhivaniye*, etc.” (Galperin, 1997, p.4)

Thus, the psyche is declared to be activity and considered through the prism of external activity. “Th at is good, but that is not quite what we set out after,” Galperin concluded, referring to the time they began moving away from Vygotsky about eight years before. As a solution, he proposed to regard the phenomena of consciousness, *perezhivaniya* as “*subjective visions*.” All such are “*not the actual, but only a former psyche*;” they are, in a sense, the Platonic shadows of real orienting activity. This is how to overcome the dualism of the external and the internal (actions and *perezhivaniya*) within human activity.

Obviously, Leontiev did not approve such a radical massacre of the inner world of consciousness. His book offered a more complex and cautious solution to the problem, namely, to depict consciousness as a structural projection of activity. On the internal mental plane, the triad *activity— action— operation* takes the form of *affect— purpose— meaning*. “In general, man’s activity is internally linked to affect, action to purpose, and operation to meaning.” (Galperin, 1997, p.4)^7^

There are two things worth noting in this formulation:

1. On the plane of consciousness, affect is activity’s double. Hence, consciousness is nothing but the *affective form* of activity.
  Vygotsky would scarcely object to that; but he could add that this is the definition of the psyche in general, not only human consciousness.
2. Meaning is correlated with *operation*; by that Leontiev removed semantics from the scope of psychology. Meaning, as operation, becomes a psychological phenomenon only when transformed into a component of the living activity of the individual.

This objection to Vygotsky was then developed and substantiated in Leontiev’s book *Activity. Consciousness. Personality*,^8^ but here, again, without mentioning the name of his opponent, Vygotsky.

In *The Basic Processes of Mental Life* (1940), Leontiev proposed a new understanding of affect as an experience (*perezhivaniye*) of the difference between the motive and the result of activity (Leontiev, 1994, p.49). The activity concept of *perezhivaniye* is elaborated in the first of his four “philosophical notebooks.” On the same pages, we find the assertion that sensation, forming the “germ cell” of psyche, is nothing but affect. “Sensation emerges as a feeling, as a vague sensation— affect.” (*Ibid*., p.164)

The thesis of the affective nature of the psyche was not covered in Leontiev’s published works, let alone systematically developed. Leontiev commented on it in his later lectures on general psychology, but without using the term “affect.” He asserted that the first, most ancient forms of sensitivity are diffuse; there is not yet a boundary between the states of a feeling body and the states of the external bodies it perceives. Differentiation between the “gnostic” and emotional functions of sensitivity “occurs slowly throughout biological evolution.” (Leontiev, 2001, p.51)

Thus, first, Leontiev actually agreed with Vygotsky that the psyche begins with affect. But in subsequent years, the term “affect” became a rare guest in his work, and the meaning of this term was narrowed to “strong, sudden emotional phenomenon.” (*Ibid*., p.462)

### Realizing the Kapital Dream

“When we want to see an oak with all its vigor of trunk, its spreading branches, and mass of foliage, we are not satisfied to be shown an acorn instead” (Hegel, 2005, p.75-76). Soviet psychologists did not get too far in realizing Vygotsky’s *Kapital* dream. The Spinozist Vygotsky came to the conclusion that the “cell” of psyche is affect. Later, Leontiev talked about “a vague sensation— affect,” in which the feeling of the object of activity with the feeling of the active body are merged.^9^ But Leontiev does not distinguish between passive and active affects, and does not admit that they determine activity. Only by reflecting itself in the affective “mirror” does the objective motive becomes the *internal, psychological* determinant of activity.

Criticizing Vygotsky’s views, Leontiev argued that “affect is not a *driving* force” (1994, p.40). One can answer with the words from the Foreword of Cannon’s book: “Fear, rage and pain, and the pangs of hunger are all primitive experiences which [...] are properly classed as among the most powerful that determine the action of men and beasts. A knowledge of the conditions which attend these experiences, therefore, is of general and fundamental importance in the interpretation of behavior.” (1922, p.vii)

Vygotsky cited these words with approval, and he undoubtedly intended to build the science of behavior on the basis of the concept of affect. Of course, we must not forget that affect is the attribute of activity. Behind affect, there stand needs on the one hand, and objective motives on the other. Affect is the effect of their interaction. As such, this interaction is studied in the sciences of material life and represents an “antediluvian” condition for the emergence of the psyche, along with the body’s morphophysiology, biochemical reactions, and so on.

Affects are the “perturbations” in the state of the body that determine its ability and readiness to act. This is also the definition of psyche in general, with the reservation that the “body” of *higher* mental forms is *objective culture*, which includes the organic body of the person, developed by human labor. Cultural, ideal affects are as different from natural affects as an orchard is from a wild wood.

The activity process consists of two opposite phases merged in each specific action: a *direct* effect upon the object and a *reflected* effect generating affects. An analysis of the phases of activity allows us to define the specificity of *psychical reflection* (see Maidansky, 2021).

What is the place and role of the psyche in the activity process? Leontiev and Galperin, for all their differences, saw this role in *orienting to the object*. This shifts the focus to the first phase of activity. However, here the psyche serves material life activity as one of its internal moments, along with simple irritability and metabolic processes. Only in the second, affective phase of activity does the psyche find its special content, different from the “physical” one. Here it forms its own “cells,” its specific domain. “The grandiose signalistics of speech” (Ivan Pavlov) are formed from the matter of emotional-expressive reactions.

As the wealth of bourgeois society consists of commodities, so the wealth of the psyche consists of affects; they are its living tissue, its flesh and blood. Affect represents, on the one hand, the wants and needs of a living being, and on the other, a sensual image or idea of an external thing (Marx liked to compare commodities with mirrors). This is, so to speak, the use-value and the exchange-value of affect.

Like commodities, affects live their own lives in processes of mutual exchange, in the element of *communication*. Here, the psyche is opposed not to the “mute” object, but to *another* psyche with its personal affects. It is necessary to deduce logically the universal forms of this interaction of “souls,” just as Marx deduced the value forms of commodity exchange in the first chapter of *Kapital*.

Empirically, it is not difficult to detect three forms of affect exchange: 1) a simple or immediate form of communication; 2) a signaling form; and 3) a language or symbolic form. The last is created by human labor and is a universal form of *cultural* activity. Vygotsky understood language in the same vein. In the language “market,” not only affect, but also thoughts and ideas are exchanged. The word succeeds in combining communication (speech) and thinking.

As early as Wolfgang Köhler, it was acknowledged that in the animal world, communication and affective life have almost no overlap with intellect and rational behaviors. Similarly, in early human childhood, speech communication develops independently of thinking. Even in adults, reason and passions are often at odds. But one fine day they conclude an alliance: thinking is poured into a word, and affect is cast into the form of a concept, becoming a cultural emotion. This is a man’s first step towards freedom.

According to Vygotsky, the evolution of mind is a process of its liberation from slavery to the passions, just as for Marx, human history is the path to the “realm of freedom” (*Reich der Freiheit*) through the Golgotha of alienated labor.

“Freedom is not given; it is taken. It is not primordial but achieved in a difficult inner struggle. Man can become free, but this is as excellent as it is rare. The path to freedom leads through steep summits. Freedom does not lie in the plain; it is not accessible and within easy reach for everyone. It lies not at the beginning but at the end of a person’s path. It is inaccessible to the child. It is not located in the depths but in the summits of the mind.” (Vygotsky, 2018, p.374) This is why Vygotsky called his unfinished project “height” or “acmeist” psychology.

Following the author of *Theses on Feuerbach*, Vygotsky might say: the psychologists have only *interpreted* human life in various ways, but the point is to *change* it. Or, as he wrote down on a scrap of paper: “To preserve life is the main function of the passions. To change life is the main function of consciousness.” (Vygotsky, 2018, p. 221)

*Das Kapital* showed us how an entire economic formation grows out of a commodity “cell.” Marxist-oriented psychology should show how the “oak” of *psychological* formation grows from the “acorn” of affect, that is, to demonstrate a series of evolutionary stages that differ from one another “by the order and connection of affects.” In this way, I believe, Vygotsky’s dream would come true, were we to take it seriously.

In Soviet psychology, formational theories were developed by Aleksei Leontiev (sensory psyche, perceptive psyche, intellect, and consciousness) and Pavel Blonsky (memory in its motor, affective, imaginary, and verbal forms). But they were empirical constructions, having no sign even of formal-geometric deduction, as in Spinoza’s *Ethica*, let alone the sophisticated dialectics of *Das Kapital*.

## Conclusion

Since the second half of the last century, the theory of activity in psychology has been developed extensively. As a “big narrative,” however, it has stagnated. “This system of notions turned out to be frozen, without any movement,” Leontiev ascertained in 1969 (1994, p.247). The perfect way to unfreeze it and set it in motion is to refer to Vygotsky’s unfulfilled plans, which his *Notebooks* have recently told us about. In his hands, activity psychology developed in a highly dynamic way.

“Orientation by the stars is most primitive. But contemporary psychology, which has no compass or map, must rely on the stars: on Spinoza.” (Vygotsky, 2018, p.221) This testament has remained the voice of one crying in the wilderness. The problem of freedom, understood as “moderating and restraining affects,” is hardly discussed in cultural-historical psychology, and the very concept of affect has been degraded.

A.N. Leontiev expressed concern about the transformation of the notion of activity into an empty abstraction. And already at the beginning of our century, his student and assistant Nina Talyzina, having condemned the fashion of renaming mental functions “activities,” summed up the disappointing result: “The activity approach has not yet been implemented. [...] We don’t have activity psychology; it should still be built. There should be an analysis in the language of actions, not in the language of functions.” (2003, p.15)

But why has it not been built? Outstanding minds worked on the development of activity psychology for a good 50 years but could not turn it into a full-fledged scienti fic system. I have no concrete answer. Perhaps its methodology, the “language of action,” appeared not powerful enough, especially in the higher spheres of mental life, such as literature and theater. The achievements of activity psychology, in its Leontiev version, became more and more modest as it moved away from the “lower boundaries.” No psychologists dream any longer of a *Das Kapital* of their own, but it may be worth a try.

## Footnotes

1 I use the term “psyche” for Russian *psykhika*, and “mind, mental” for the *human* psyche or the higher (cultural) psychological functions, in Vygotsky’s sense.

2 In the English edition of Vygotsky’s *Educational Psychology*, “*aktivnost”* is rendered as “purposefulness,” *psikhicheskiy* as “passive,” etc. I corrected the terms in quotations.

3 The title of the Russian edition (1927) of Cannon, 1920.

4 The paper has not yet been published; it is quoted in Zavershneva, 2009, p.130.

5 In Russian, *vershinnaja psikhologia*. The interpreters have not yet agreed on how best to translate it. Literally, *vershina* means a peak of a tree or a mountain. In his notebooks, Vygotsky uses the synonymous expression “acmeistic psychology.” Acmeism was a realistic trend in Russian poetry that was sharply opposed to symbolism; Mandelshtam defined acmeism as the “longing for world culture.” And Vygotsky contrasts *vershinnaja psikhologia* with *Tiefenpsychologie* (depth psycho logy), which explains human behavior and culture by unconscious processes.

6 Leontyev’s first address to this topic took place back in his student years, at the suggestion of his supervisor Georgi Chelpanov. Then, along with Aleksander Luria, they studied “objective symptoms of affective reactions” with the apparatus for recording disorders in fine motor movements.

7 This passage from Leontiev’s *Development of the Psyche* is cited verbatim in Galperin’s letter.

8 “Meanings and the operations, contained within them, *in themselves*, that is, in their abstraction from internal relations of the system of activity and consciousness, are not at all the subject of psychology.” (Leontyev, 2004, p. 111)

9 Vygotsky called this kind of vague sensation “affective perception,” in which “emotions and perceptions are not yet differentiated between them.” This psychological function is formed in the early childhood first and occupies “the dominant place in relation to all the others” (2001, p. 98–99).
